# Assessment of standard precaution related to infection prevention readiness of healthcare facilities in Bangladesh: Findings from a national cross-sectional survey

**DOI:** 10.1017/ash.2021.226

**Published:** 2021-12-09

**Authors:** Md Abdullah Al Jubayer Biswas, Md Zakiul Hassan, Mohammad Riashad Monjur, Md Saiful Islam, Aninda Rahman, Zubair Akhtar, Fahmida Chowdhury, Sayera Banu, Nusrat Homaira

**Affiliations:** 1 Programme for Emerging Infections, Infectious Disease Division, International Centre for Diarrhoeal Disease Research, Bangladesh (icddr,b), Mohakhali, Dhaka, Bangladesh; 2 Nuffield Department of Medicine, University of Oxford, Oxford, United Kingdom; 3 Faculty of Health and Medicine, University of Newcastle, Callaghan, New South Wales, Australia; 4 School of Public Health and Community Medicine, University of New South Wales, Sydney, Australia; 5 Communicable Disease Control (CDC), Directorate General of Health Services, The Ministry of Health & Family Welfare, Government of Bangladesh, Dhaka, Bangladesh; 6 School of Women’s and Children’s Health, Faculty of Medicine, University of New South Wales, Sydney, New South Wales, Australia; 7 Respiratory Department, Sydney Children’s Hospital Randwick, Randwick, New South Wales, Australia

## Abstract

**Background:**

Baseline assessment of standard precaution relating to infection prevention and control (IPC) preparedness to fight health crisis within healthcare facilities at different levels and its associated factors in Bangladesh remains unknown.

**Methods:**

We analyzed the nationally representative Bangladesh health facility survey (BHFS) data conducted by the Ministry of Health and Family Welfare (MoHFW) during July–October 2017. We used the World Health Organization (WHO) Service Availability and Readiness Assessment (SARA) manual to determine the standard precautions related to the IPC readiness index. Using a conceptual framework and multivariable linear regression, we identified the factors associated with the readiness index.

**Results:**

We analyzed data for 1,524 surveyed healthcare facilities. On average, only 44% of the standard precaution elements were available in all facilities. Essential elements, such as guidelines for standard precautions (30%), hand-washing soap (29%), and pedal bins (38%), were not readily available in all facilities. The tuberculosis service area was least prepared, with 85% of elements required for standard precaution deficient in all facilities. Significantly lower readiness indexes were observed in the rural healthcare facilities (mean difference, −13.2), healthcare facilities administered by the MoHFW (mean difference, −7.8), and private facilities (mean difference, −10.1) compared to corresponding reference categories.

**Conclusions:**

Our study revealed a severe lack of standard precaution elements in most healthcare facilities, particularly in rural health centers. These data can provide a baseline from which to measure improvement in infection prevention and control (IPC) in these facilities and to identify areas of gaps for targeted interventions to improve IPC strategies that can improve the Bangladesh health system.

Although a tremendous amount of progress has been achieved in expanding healthcare access in low- and middle-income countries (LMICs) over the last 20 years, the capacity and preparedness of healthcare facilities to ensuring safe and effective healthcare delivery have received limited attention.^1^ In 2019, the World Health Organization (WHO) estimated that 134 million adverse events annually, including 2.6 million avoidable deaths, were attributable to unsafe care provided by healthcare facilities in LMICs.^2^ Of these adverse events, healthcare-associated infections (HAIs) were the most frequent, with the average prevalence of HAIs in LMICs (ie, 15.5%) double that in high-income countries such as Europe (7.1%) and the United States (4.5%).^3,4^

Standard precautions related to infection prevention and control (IPC) are fundamental to curtailing HAIs by creating a safe care environment; they reduce the risk of cross transmission of infections and ensure personal safety in the healthcare setting.^5^ In implementing such processes, LMICs face significant challenges of overcrowding, suboptimal understanding of hygiene and sanitation, logistical bottlenecks, inadequate surveillance and laboratory capacity, and a lack of trained professionals.^6,7^ These health-system challenges severely constrained the ability to limit transmission of COVID-19 pandemic in LMICs, which primarily spread via respiratory droplets and direct contact.^7^ As noted by the WHO interim guideline of IPC in the context of health care for COVID-19, applying standard precautions for suspected and diagnosed cases of COVID-19 is a core approach to healthcare infection control.^8^

We conducted a comprehensive assessment of standard precaution readiness related to infection prevention within healthcare facilities to identify gaps and opportunities to strengthen infection control preparedness in the healthcare setting. We further explored associations between standard precaution readiness and healthcare facility infrastructure and system characteristics. Our results provide important baseline information to policy makers in resource-limited countries such as Bangladesh.

## Methodology

### Data source and procedures

We performed and analysis of secondary data from the Bangladesh health facility survey (BHFS) 2017, which are publicly available. The survey was carried out between July and October 2017 by the National Institute of Population Research and Training (NIPORT) under the Ministry of Health and Family Welfare (MoHFW). The purpose of the survey was to obtain data on the different kinds of healthcare facilities in Bangladesh and the overall quality of health of treatments offered by them. The team adopted the survey tools from the Demographic and Health Surveys (DHS) Program of the US Agency for International Development (USAID), as used in the service provision assessment component, and standardized it after consulting with the Directorate General of Health Services, the Directorate General of Family Planning Services, and other key stakeholders.^9^ The final survey report detailed the survey design and survey implementation.^9^ The survey data collection tools were broadly classified into 2 core components: facility inventory and healthcare provider tools. The tools covered information related to service availability, general facility readiness, service-specific readiness, and healthcare-provider training and education. Trained data collectors observed the availability of the specific items, including the facility infrastructure, and they recorded information using a handheld tablet computer. We extracted data on standard precautions for infection prevention readiness from facility inventory data. We used the Service Availability and Readiness Assessments (SARA) tools jointly developed by the World Health Organization (WHO) and the United States Agency for International Development (USAID). The tool included the following elements of standard precautions: facility waste management, availability of disinfectant, soap, running water, guidelines for standard precaution, pedal bins/plastic bins, safety boxes, standard disposable syringes with needles, and disposable latex gloves.^10^

### Sample size and sampling process

We calculated the sample size using the following formula: n = (1 − *p*)/ϵ^2^ *p*, where ϵ was the requested survey precision, which is the relative standard error for estimating a proportion (*p*).^9^ A sample of 1,600 health facilities was calculated for the BDHS survey by controlling the relative standard error for an indicator at the 30% level (*p* = 0.30) within 15% (ϵ = 0.15) at the domain level and within 20% (ϵ = 0.20) at the oversampled district level, and by ensuring that the survey precision is comparable across divisions.^9^ The study sites were identified using a sample framework consisting of a list of 19,811 registered healthcare facilities. The MoHFW provided the list of registered healthcare facilities. The total sample of 1,600 healthcare facilities was dispersed across sampling domains, including healthcare facilities type, management authority, division, and over sample districts. Information on 1,524 healthcare facilities was included in the final data set because the rest of the facilities were either closed or inactive when the survey was conducted. The survey contained a sample of all varieties of registered healthcare facilities in all 8 divisions. The BHFS 2017 covered all 6 categories of public healthcare facilities and community clinics (CCs, n = 331), union subcenters and rural dispensaries (USCs/RDs, n = 216), union health and family welfare centers (UHFWCs, n = 497), Upazila health complexes (UHCs, n = 141), mother-and-child welfare centers (MCWCs, n = 91), and district hospitals (DHs, n = 62), as well as nongovernmental organization clinics and hospitals (NGOs, n = 130) and private hospitals (PHs, n = 132). The survey report contains further details on each type of healthcare facility sampling technique and management structure.^9^ Sample distributions by division and health facility type are depicted in Fig. 3.

### Standard precaution readiness index calculation

We extracted variables relating to the DHS service provision assessment matching SARA manual recommendation.^9,10^ We recorded measurement tracer items, binary components used to assess readiness, according to Table 1. We calculated the percentage score by taking the sum and means of the tracer items by facilities based on SARA guidelines in each service domain, which indicates the average required availability of tracer items for standard precaution at each service delivery domain.^10,11^ In each healthcare facility, 9 service delivery domains were studied: the general outpatient care area, child and adolescent vaccination services area, child growth monitoring area, family planning area, antenatal care services (ANC) and postnatal care service area (PNC), delivery and newborn care area, tuberculosis care area, noncommunicable care area, and waste management. The arithmetic mean of the resulting scores was then calculated for a standard precaution readiness index of each service domain of all the healthcare facilities. Similarly, the overall percentage of the score was calculated considering each value of the index tracer items of all 9 service domains of all healthcare facilities. Mean scores were calculated to show the average required accessibility of tracer items in the 9 service delivery domains.

**Table 1.** Measurement of Tracer Items of Standard Precaution Readiness Index and Explanatory Variables

Variables	Explanation
**Tracer item**	
Disposal of sharps waste like filled sharps boxes	The composite variable reflected type of sharps waste management including the burn-in incinerator, open burning, dump without burning, remove offside and never have sharps waste; grouped into 1 = yes (burn-in incinerator or open burning or dump without burning or remove offside) and 0 = no (never have sharps waste or other)
Disposal of medical waste other than sharps boxes	Medical waste disposal factor other than sharps box was recoded as like sharps waste disposal factor.
Sharps container/safety box	The observation about the availability of sharps containers in all the service delivery areas of a facility was grouped into 1 = yes (observed or reported not seen), 0 = no (not available)
Waste receptacle (pedal bin) with lid and plastic bin liner	Investigation about the presence of the waste container in all the care delivery areas of a facility. It was grouped “observed” or “reported not to seen” into “1 = yes” and “not available” into “0 = no.”
Disinfectant/antiseptics like chlorine, hibitane, alcohol	Examination of the convenience of disinfectant in all units of a facility. It recorded as 1 = yes, for observed or reported not seen and 0 = no for not available
Single-use standard disposable syringes with needles or auto-disable syringes with needles	Observation on having auto-disable syringes with needles in all care delivery areas of a facility and recoded as 1 = yes, for observed or reported not seen and 0 = no for not available
Running water and hand-washing soap or alcohol-based hand rub	Observation on availability of running water, handwashing soap, alcohol-based hand rub in all the required area of the facility and recoded as 1 = yes, for observed or reported not seen and 0 = no for not available
Disposable latex/other gloves	This composite variable represented observation of availability of disposable latex and grouped into 1 = yes (observed or reported not seen), 0 = no (not available)
Guidelines for standard precautions	Observation on availability of guidelines for standard precautions in the specific area of the facility and recoded as 1 = yes, for observed or reported not seen and 0 = no for not available
**Explanatory variables**	
Type of health facility	8 different types of health facilities: district hospital (DH), mother and child welfare center (MCWC), Upazila health complex (UHC), union health and family welfare centre (UNFWC), union subcenter (USC), community clinic (CC), nongovernmental organization (NGO) clinic or hospital, and private hospital (PH)
Location	Types of healthcare facility locations: urban and rural
Division	The provincial location of healthcare facilities at the time of the survey: Barishal, Chattogram, Dhaka, Khulna, Mymensingh, Rajshahi, Rangpur, Sylhet
Managing authority	Ownership of the healthcare facility at the time of the survey: government (MoHFW), local government under the local government division (LGD), NGO, private for-profit

### Assessment of explanatory variables

We estimated the standard precaution readiness index and investigated it according to various explanatory variables, including the type of healthcare facilities, location, division, the managing authority, and supervision from the higher authority. Explanatory variables were identified based on the existing literature.^9,12,13^ The explanatory variables are explained in Table 1.

### Statistical analysis

**Fig. 1:**
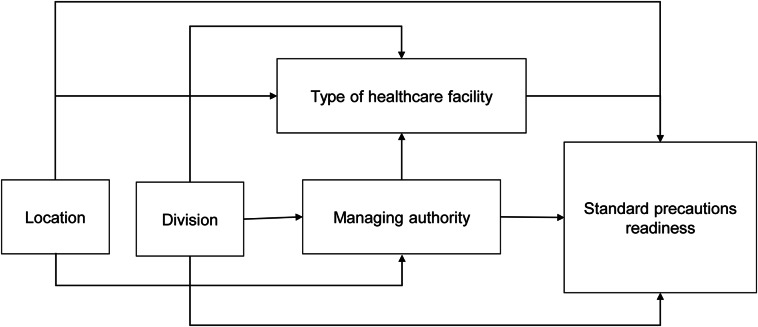
A conceptual framework for potential factors associated with standard precaution readiness index, 2017 Bangladesh.

We conducted the descriptive analysis using frequency, percentage, arithmetic mean, standard deviation (SD), horizontal bar graph, and 95% confidence interval (CI) to summarize healthcare facilities used in the survey and standard precaution readiness index score according to different healthcare-facility characteristics. We also used linear regression to compare the standard precautions readiness indices according to explanatory variables, and we reported them as the coefficient with the 95% CI. We then utilized the conceptual framework technique to delineate the causal relationship between readiness score and explanatory variables, which helped determine the potential factors associated with the readiness index.^14,15^ Specialized points of interest in the conceptual framework technique have been clarified by Jewell et al.^16^ Here, we depicted the specific application for associated factors of readiness index (Fig. 1). According to our framework, for example, for the type of healthcare facility, we identified location, division, and managing authority as causal factors, and we adjusted these causal factors in the multivariable linear regression analysis.^12,13^ Similarly, we carried out multivariable linear regression for other explanatory variables.^9,17–19^ Outputs of multivariable linear regression were reported as adjusted coefficients with a 95% CI. All descriptive, bivariable, multivariable analyses were performed considering survey sampling design, sampling strata, and weight. We considered a *P* of .05 or lower to indicate statistically significant difference.

### Ethics approval and consent to participate

The 2017 Bangladesh health facility survey data are publicly available. We were able to download the DHS program data from the DHS website (https://dhsprogram.com/data/available-datasets.cfm). According to the DHS, written informed consent was received from the manager or the person in charge of the responsible most senior health worker before participation in the survey. Confidentiality was maintained during data collection. Ethical approval for this study was not needed because it was based on publicly available secondary data.

## Results

**Fig. 2:**
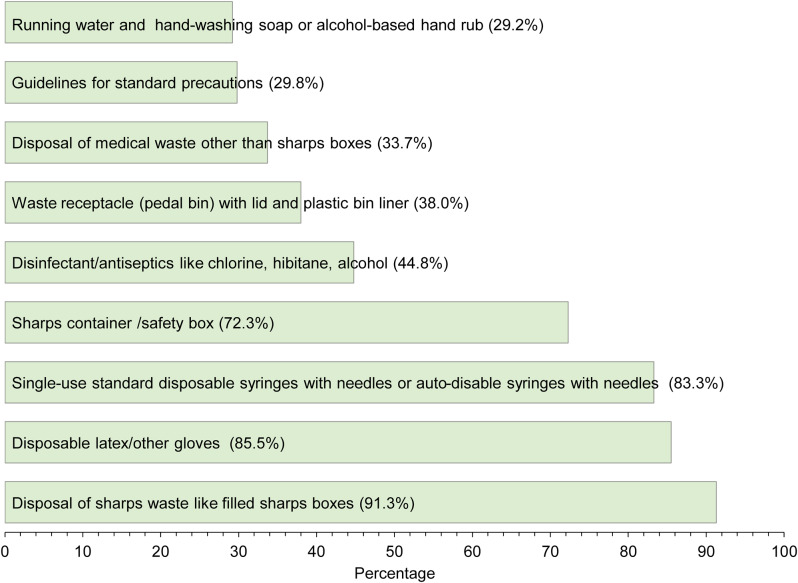
Percentage distribution of tracer items of standard precaution related to infection prevention in sampled healthcare facilities, 2017 Bangladesh.

We included data on standard precautions of 1,524 healthcare facilities in our analysis. Among 1,524 healthcare facilities, 1,012 (66.4%) were community clinics, and 5 were large district hospitals (0.3%). The vast majority of facilities were located in rural areas (n = 1,416, 92.9%), while the Dhaka division had 304 (19.9%) facilities. Most healthcare facilities were public facilities (93.0%) under the MoHFW of Bangladesh; 53 (3.5%) were operated by a nonprofit organization; and 43 (2.8%) were private facilities (Table 2). As illustrated in Fig. 2, the availability of tracer items of standard precautions across these 1,524 facilities was limited, particularly the availability of running water and hand-washing soap or alcohol-based hand rubs (29.2%), pedal bins (38%) and guidelines for standard precautions (29.8%), and disposal of medical waste (33.7%).

**Table 2.** Distribution of Surveyed Healthcare Facilities by Background Characteristics, 2017 Bangladesh (N = 1,524)

Background Characteristics	Unweighted No.	Weighted No.	Weighted %
Total	1,524	1,524	100.0
**Type of facility**			
District hospital (DH)	62	5	0.3
Mother and child welfare centre (MCWC)	90	7	0.5
Upazila health complex (UHC)	141	32	2.1
Union health and family welfare centre (UNFWC)	479	250	16.4
Union subcenter (USC)	198	111	7.3
Community clinic (CC)	325	1012	66.4
Nongovernmental organization (NGO) clinic/hospital	123	64	4.2
Private hospital (PH)	106	43	2.8
**Location**			
Urban	383	108	7.1
Rural	1141	1416	92.9
**Division**			
Barishal	260	113	7.4
Chattogram	302	288	18.9
Dhaka	188	304	19.9
Khulna	164	187	12.3
Rajshahi	161	220	14.4
Rangpur	158	193	12.7
Sylhet	163	96	6.3
Mymensingh	128	123	8.0
**Managing authority**			
Government (MoHFW)	1295	1418	93.0
Local government	17	11	0.7
NGO	106	53	3.5
Private for profit	106	43	2.8

**Fig. 3:**
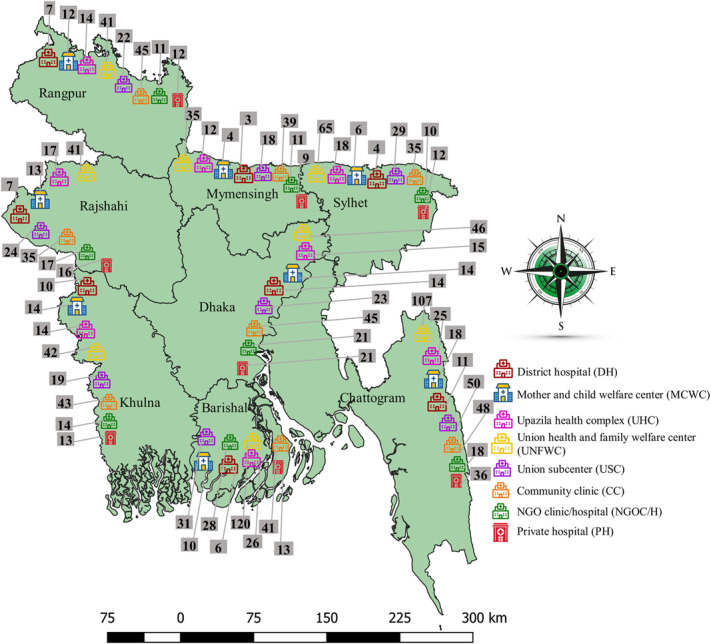
Distribution of sampled healthcare facilities by division and healthcare facilities’ type, 2017 Bangladesh.

### Readiness index of standard precautions for infection control

Fewer than half (44%) of the tracer items required for standard precaution at 9 service delivery domains were available in the 1,524 healthcare facilities. Regarding each care service domain, the average required availability of basic amenities of standard precaution ranged from 15% in tuberculosis care areas to 63% in the waste management domain (Table 3). The average standard precaution readiness index also varied from 36% for USCs to 72% for DHs (Table 3).

**Table 3.** Standard Precaution Readiness Index According to Different Characteristics of the Healthcare Facilities, 2017 Bangladesh

Characteristics of the Healthcare Facilities	Standard Precaution Readiness Index (%)	
	Mean±SD	95% CI
Among all facilities	44.3±19.0	(42.3–46.3)
**Service area of the healthcare facility**		
The general outpatient area where most client services	57.9±23.5	(55.6–60.2)
Child and adolescent vaccination services	52.3±29.1	(50.0–54.6)
Child growth monitoring services	56.8±24.3	(54.8–58.8)
Family planning services	53.5±28.0	(50.7–56.3)
Antenatal care services, postnatal care service	57.8±23.6	(55.6–60.0)
Delivery and new-born care service	16.4±31.5	(12.8–20.0)
Tuberculosis services	14.8±28.6	(11.8–17.8)
Noncommunicable or chronic diseases service	39.5±31.0	(35.1–43.9)
Waste management	62.5±24.6	(60.4–64.6)
**Type of facility**		
District hospital (DH)	72.0±76.4	(65.5–78.5)
Mother and child welfare center (MCWC)	57.1±59.0	(53.1–61.1)
Upazila health complex (UHC)	69.9±38.6	(65.2–74.7)
Union health and family welfare center (UNFWC)	49.9±24.1	(46.2–52.6)
Union subcenter (USC)	36.0±30.8	(32.9–39.2)
Community clinic (CC)	40.9±9.5	(38.5–43.5)
Nongovernmental organization (NGO) clinic/hospital	62.6±23.8	(58.2–66.9)
Private hospital (PH)	58.7±26.8	(52.2–65.2)
**Location**		
Urban	63.7±34.1	(61.1–66.4)
Rural	42.8±16.4	(40.8–44.7)
**Division**		
Barishal	45.2±28.1	(39.0–51.2)
Chattogram	44.9±17.1	(38.3–51.6)
Dhaka	43.6±15.0	(35.8–51.6)
Khulna	45.8±17.0	(42.5–49.1)
Rajshahi	49.1±18.9	(45.0–53.2)
Rangpur	40.6±18.4	(37.9–43.3)
Sylhet	43.0±22.1	(37.5–48.5)
Mymensingh	39.1±16.3	(36.0–42.1)
**Managing authority**		
Government (MoHFW)	43.0±17.7	(41.0–45.0)
Local government	68.7±18.0	(64.6–72.9)
NGO	61.3±24.6	(56.2–66.4)
Private for profit	58.7±26.8	(52.2–65.2)

### Factors associated with the readiness index specific to standard precautions for infection control

Table 4 shows the bivariable and multivariable linear regression analysis of the standard precaution readiness index of all nine areas of the 1,524 healthcare facilities with facility characteristics. In the adjusted model, all other types of facilities had a significantly lower standard precaution readiness index than district hospitals. The USCs experienced the largest significant decrease (mean difference, −28.9; 95% CI, −36.2 to −21.5), and MCWCs experienced the smallest significant decrease (mean difference, −13.2; 95% CI, −19.9 to −6.4). The standard precaution index of rural healthcare facilities significantly differed from that of urban healthcare facilities, with a factor of 19.2 (95% CI, −22.3 to −16.2). Similarly, private facilities and facilities under MoHFW had a lower precaution readiness index compared to facilities under the local government division (LGD), with an estimated mean difference of a factor of 7.8 (95% CI, −11.9 to −3.6) and 10.1 (95% CI, −21.2 to −13.4), respectively.

**Table 4.** Bivariable and Multivariable Linear Regression Analysis to Investigate Associated Factors, Bangladesh, 2017

Characteristics of the Healthcare Facilities	Unadjusted Model			Adjusted Model		
	Estimated Mean Difference (95% CI)	*P* Value	R^2^	Estimated Mean Difference (95% CI)	*P* Value	R^2^
**Type of facility^a^**						
Mother and child welfare centre (MCWC)	−14.9 (−22.5 to −7.3)	**<.006**	0.15	−13.2 (−19.9 to −6.4)	**<.001**	0.15
Upazila health complex (UHC)	−2.1 (−10.0 to 5.9)	1.000	0.15	0.3 (−7.6 to 7.1)	.942	0.15
Union health and family welfare centre (UNFWC)	−22.1 (−29.1 to −15.1)	**<.001**	0.15	−16.1 (−23.4 to −8.7)	**<.001**	0.15
Union subcenter (USC)	−36.0 (−43.1 to −28.8)	**<.001**	0.15	−28.9 (−36.2 to −21.5)	**<.001**	0.15
Community clinic (CC)	−31.0 (−38.0 to −24.1)	**<.001**	0.15	−24.3 (−31.6 to −17.0)	**<.001**	0.15
Nongovernmental organization (NGO) clinic/hospital	−9.5 (−17.2 to −1.7)	.494	0.15	2.9 (−6.7 to 12.5)	1.000	0.15
Private hospital (PH)	−13.3 (−22.4 to −4.2)	.138	0.15	3.5 (−15.1 to 22.2)	1.000	0.15
District hospital (DH)	Reference			Reference		
**Location^b^**						
Rural	−21.0 (−24.2 to −17.6)	**<.001**	0.08	−21.0 (−24.2 to −17.6)	**<.001**	0.08
Urban	Reference			Reference		
**Division^b^**						
Chattogram	−0.2 (−10.0 to 9.4)	1.000	0.02	−0.2 (−10.0 to 9.4)	1.000	0.02
Dhaka	−1.6 (−12.3 to 9.1)	1.000	0.02	−1.6 (−12.3 to 9.1)	1.000	0.02
Khulna	0.5 (−6.9 to 8.0)	1.000	0.02	0.5 (−6.9 to 8.0)	1.000	0.02
Rajshahi	3.9 (−4.0 to 11.8)	1.000	0.02	3.9 (−4.0 to 11.8)	1.000	0.02
Rangpur	−4.6 (−11.7 to 2.4)	1.000	0.02	−4.6 (−11.7 to 2.4)	1.000	0.02
Sylhet	−2.2 (−11.1 to 6.6)	1.000	0.02	−2.2 (−11.1 to 6.6)	1.000	0.02
Mymensingh	−6.2 (−13.5 to 1.1)	1.000	0.02	−6.2 (−13.5 to 1.1)	1.000	0.02
Barishal	Reference			Reference		
**Managing authority^c^**						
Government (MoHFW)	−25.7 (−30.3 to −21.1)	**<.001**	0.06	−7.8(−11.9 to −3.6)	**<.001**	0.10
NGO	−7.4 (−14.1 to −0.7)	**<.001**	0.06	0.9(−4.4 to 6.2)	.738	0.10
Private for profit	−10.0 (−17.7 to −2.3)	**<.001**	0.06	−10.1(−16.6 to −3.5)	**.003**	0.10
Local government	Reference			Reference		

a Multivariable model adjusted for location, division, managing authority.

b Multivariable model not adjusted with any covariate.

c Multivariable model adjusted for location, division.

## Discussion

In this comprehensive national assessment of service provision of healthcare facilities, we found Bangladesh’s healthcare facilities were grossly deficient, with <50% (maximum score, 100%) of the basic infrastructure and tools needed for standard precautions for infection prevention available in all healthcare facilities. Although our overall readiness finding of 44% was similar to estimates of infection prevention scores from local studies in Bangladesh as well as analysis in Ethiopia (48%), none of these specifically analyzed standard precautions, which is of particular importance to evaluate the preparedness of healthcare facilities in the context of a pandemic.^20–23^ Thus, our analysis provides baseline data to monitor progress over time.

Of all service areas, tuberculosis services were the least prepared, with 85% of the essential components of standard precaution unavailable in facilities, suggesting the possibility of nosocomial transmission of droplet infection. Other studies have also identified adoption and implementation of tuberculosis IPC methods in Bangladeshi healthcare settings.^24^ Our findings highlight that implementing national tuberculosis (TB) infection prevention and control strategies with particular emphasis on preventing droplet transmission may help reduce nosocomial infection of not only TB but also other infectious diseases during disease outbreaks.

We have demonstrated that healthcare facilities in rural Bangladesh were significantly less prepared compared to urban facilities, with a 21.0% lower readiness score (95% CI, 24.2–7.6). Notably, >60% of Bangladesh’s total population reside in rural areas. Although our analysis was not controlled for the type of facility, this may partly be explained by Bangladesh’s tiered health system with the predominance of community clinics in rural regions, which we found had a 31% lower readiness score in standard precautions compared to tertiary-care district hospitals in urban cities.^25^ This finding was consistent with a previous analysis in other LMICs, which also could be attributed to the differences in the distribution of healthcare facilities between urban and rural areas.^12^ This situation is problematic given that community clinics account for 66.4% of healthcare facilities (n = 1,012) in the country, whereas district hospitals account for merely 0.3% (n = 5). Therefore, the readiness score in community clinics is a more holistic reflection of the readiness of Bangladesh and, in particular, rural Bangladesh.

The administrative authority of the facilities in our analysis had a significant impact on the readiness score of healthcare facilities. Those managed by the ministry of local government were significantly better equipped than facilities managed by the MoHFW (−25.7%); NGOs (−7.4%); and private institutions (−10.0%). This difference may be attributed to the public–private partnership (PPP) model implemented by the local government division of Bangladesh to provide care for financially disadvantaged rural dwellers in the capital cities and 4 district cities.^9^ PPP entails collaborative agreements between 2 or more agents of the public and private sectors to work together to achieve a common goal.^26^ The PPP model to provide healthcare illustrates a positive effect on health outcome indicators in several countries driven by utilizing supply chain management and reduction in operating and construction costs.^27^

This study is the first to investigate the readiness of standard precaution at all levels of healthcare facilities, comprising 1,524 sampled facilities using the latest national representative service provision assessment survey data. The survey was conducted before the COVID-19 pandemic. Thus, the findings of this study could be considered a baseline assessment of standard precaution readiness in Bangladesh. However, our study includes certain notable limitations inherent to the analysis of secondary data. Data were missing for some of the standard precaution items at the service delivery area level and were not included in the analysis. We were unable to analyze healthcare-provider–related factors in this study because standard precautions data specific to healthcare providers were not available in the service provision assessment survey.

The coronavirus disease 2019 (COVID-19) pandemic has raised concerns about the preparedness of healthcare facilities in LMICs to combat not only emerging threats but also ongoing endemic diseases. Our data indicate that healthcare facilities in Bangladesh experience a shortage of essential standard-precaution resources related to IPC. These shortages are most significant in healthcare facilities located in rural areas and in the MoHFW-administered primary-care facilities. The findings of this study will aid policy makers and stakeholders in focusing on the availability of appropriate resources, designing evidence-based interventions, improving accountability and sustainability regarding standard precaution and overall IPC to help the healthcare system better prepare to respond to emerging health threats.
